# Structural Insights into Ankyrin Repeat-Containing Proteins and Their Influence in Ubiquitylation

**DOI:** 10.3390/ijms22020609

**Published:** 2021-01-09

**Authors:** Emma I. Kane, Donald E. Spratt

**Affiliations:** Gustaf H. Carlson School of Chemistry and Biochemistry, Clark University, 950 Main St., Worcester, MA 01610, USA; emkane@clarku.edu

**Keywords:** ankyrin repeat, ubiquitin, ubiquitylation, E3 ubiquitin ligases, deubiquitylase, cancer development

## Abstract

Ankyrin repeat (AR) domains are considered the most abundant repeat motif found in eukaryotic proteins. AR domains are predominantly known to mediate specific protein–protein interactions (PPIs) without necessarily recognizing specific primary sequences, nor requiring strict conformity within its own primary sequence. This promiscuity allows for one AR domain to recognize and bind to a variety of intracellular substrates, suggesting that AR-containing proteins may be involved in a wide array of functions. Many AR-containing proteins serve a critical role in biological processes including the ubiquitylation signaling pathway (USP). There is also strong evidence that AR-containing protein malfunction are associated with several neurological diseases and disorders. In this review, the structure and mechanism of key AR-containing proteins are discussed to suggest and/or identify how each protein utilizes their AR domains to support ubiquitylation and the cascading pathways that follow upon substrate modification.

## 1. Introduction

Many proteins have evolved through gene duplication and recombination events to produce repetitive motifs in their primary sequences. These non-overlapping repeat regions, commonly referred to as tandem repeats, provide a high amount of sequence conservation that are generally thought to prevent deleterious residue substitutions that cause alterations to the global fold of the domain. Another tangible benefit of tandem repeats can provide multiple binding sites for various intracellular proteins that can play an important role in protein structural integrity. Tandemly occurring repeats within the primary sequence display specific characteristics in their three-dimensional structure, forming an integrated assembly which allows for tandem repeat domain characterization [1]. The classification of tandem repeats is based upon the formation and specific localization of secondary structural elements such as α-helical bundles, β-hairpin loops, β-sheets and propellers, and horse shoe shapes [2,3,4]. Although identification of tandem repeats relies heavily on these characteristics, the size and diversity can greatly vary. Intriguingly, tandem repeat domains occur in 14% of all eukaryotic proteins and are three times as likely to occur in eukaryotic than prokaryotic proteins [5]. Four abundant classes of repeat domains exist that vary in their elongated structures that facilitate protein–protein interactions that include ankyrin repeats (AR), leucine rich repeats (LRR), armadillo repeats (ARM), and tetratricopeptide repeats (TPR) [6]. Each repeat domain acts as a scaffold for substrate proteins and their selectivity is dependent on the subtle differences in primary sequence within each repeated domain. It is unclear which residues are critical for the scaffolding structure and which are required for the overall function of the domain [7]. While protein–protein interactions are regulated through specific amino acid sequences or structural characteristics, variations within the surface exposed residues in AR domains enable specific protein binding [6].

### 1.1. Ankyrin-Repeat Containing Proteins

AR domains were first discovered as a repeating sequence in *Saccharomyces cerevisiae* cell cycle regulators Swi6, cell division control protein 10 (Cdc10) and Notch in *Drosophila melanogaster* [8]. This ~33-amino acid long repeat subsequently was named after the cytoskeletal protein ANKYRIN, a 206 kDa protein that contains 24 tandem repeats [9]. Since its initial discovery, AR domains have been observed to be present in many eukaryotic proteins, making this domain potentially the most abundant repeat domain in the eukaryotic proteome [7]. To date, there are over 367,000 predicted AR domains found within 68,471 nonredundant proteins annotated in the SMART database [10,11]. With such prevalence of AR-containing proteins in eukaryotes coupled with AR domains acting as scaffolds to facilitate protein–protein interactions in the cell, it is speculated the AR domain originated through evolutionary pressure events to provide the necessary function of facilitating the variety of signaling pathways eukaryotic organisms use to regulate cellular homeostasis [6]. 

Comparing AR-containing proteins has proven to be difficult as each protein has acquired various characteristics through multiple evolutionary events. This is largely due to conservation within AR domains relying on various residue types rather than requiring highly conserved residues at specific sites. Given that specific residue types influence the protein’s secondary structure coupled with AR domains not relying on specific residue conservation, it suggests the AR domain is defined primarily on its 3D structural fold rather than its functional support for AR-containing proteins. For example, highly conserved regions of AR domains exist between each repeat, whereas variation of hydrophobic residues can occur while maintaining the structural integrity of the domain [12]. Conserved motifs that influence α-helical and β-loop folds have recently been identified in AR domains has allowed for better AR domain identification and prediction from the primary sequence of a protein. 

**Figure 1:**
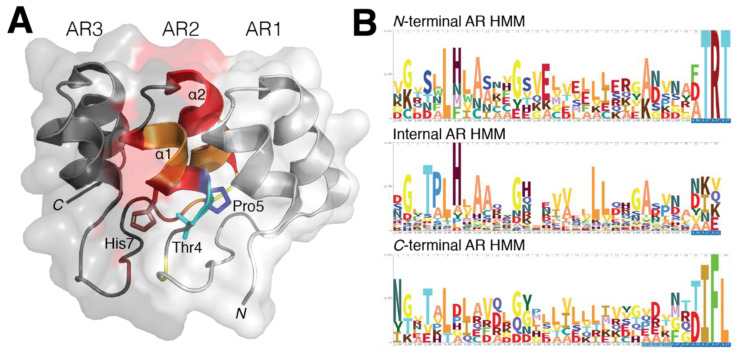
Structural conservation within ankyrin repeat domains. (**A**) Residues Thr4, Pro5 and His7 play a critical role in providing both the 90° and L-shape formation through hydrogen bonding. The elongated solenoid shape of an AR domain is predominantly dictated by these three residues while hydrophobic interactions in the core of the AR are required to stabilize the domain’s 3D fold. (**B**) The hidden Markov model (HMM) profile of the AR-containing proteins for *N-*terminal, internal and *C*-terminal repeats were analyzed to highlight the occurrence of residues in identified AR domain families. The classic G-X-TPLHLA motif was readily identified to have a strong probability of occurring in these AR-containing proteins, whereas in both *N*- and *C*-terminal repeats were observed to contain only portions of this motif. While conservation within AR domains is more prevalent within the internal and *C-*terminal repeats, the *N-*terminal AR still retains similar AR domain characteristics.

In comparison to naturally occurring AR domains, there is little deviation from its typical helix–loop–helix–β–hairpin/loop structure, which is supported through conservation of residue type. Inter-domain interactions tend to be short distances, which aids in the linear solenoid packing the AR domain fold rather than a typical globular shape. Both hydrophobic interactions through non-polar regions of the inter and intra α-helices as well as hydrogen bonding through polar residues found near the N-terminus define and stabilize the AR domain’s structural integrity [6,13] (Figure 1A).

While the hydrophobic interaction between residues Pro5 and His7 influences the domain’s L-shape, His7 also interacts with residue Thr4 through hydrogen bonding to define the 90° fold with each additional repeat [6]. The AR domain also requires nonpolar residues on surface α-helices, specifically at residues 6, 8, 9, 10, 17, 20–22, to limit solvent accessibility [12], although some variability in these polar residues of *N*- and *C*-terminal AR domains has been observed. Recent insights in AR domain conservation has identified specific residues and motifs frequently occupying the primary sequence of an AR domain. This includes the identification of a G-X-TLPHLA motif and two conserved glycine residues that allow for antiparallel helix termination and β-hairpin loop formation. Throughout the ~33 amino acids, probability of conserved residues to occur within eukaryotic AR-containing proteins remains relatively high, with an increase in conservation within the mid-region (Figure 1B).

While tandem repeats are likely to avoid detrimental mutations deriving from evolutionary events, the AR domain has unique characteristics to its mutation potential. Addition or substitutions within the primary sequence are tolerated and reported to occur in 9% of natural AR-containing proteins, while the structural integrity of an AR domain is sensitive to any residue deletions [14]. The plasticity of the AR domains remains intriguing, considering additions within the primary sequence can range from a single residue upwards to a separate folded domain as observed in TRABID [15]. It is noteworthy such additions typically reside within the loop region preceding each β-hairpin [6]. Observations in intact AR domains with deletions, on the other hand, tend to be within the antiparallel α-helices to cause helical shortening. This is evident in the cyclin-dependent kinase inhibitor (CKI) INK4 family of proteins that contain a shortened inner helix brought on by deletions within the second AR [6]. This variety of insertion size can cause the improper annotation of AR domain evaluation through various databases due to the misinterpretation of the primary sequence, thus resulting in the presence of AR domains that may go unnoticed. 

To better understand AR domain folding, researchers have recently synthesized generic two, three and four AR domain protein constructs. These sequences were derived from approximately 4000 AR domain-containing protein sequences while ensuring general conservation within the identified AR motifs [7]. Their studies support the notion that the conservation levels of residues dictate the AR domain structure and function. For example, the β-hairpin/loop region and short α-helix, which typically acts as the protein recognition site, remains semiconserved in comparison to the convex surface residues being highly conserved [7]. The innate flexibility of the AR domain recognition site allows for a multitude of binding partners, whereas the highly conserved regions act as the structural backbone to serve as a scaffold in support of the recognition site. Further evaluation utilizing generic AR constructs will aid in the elucidation of the unique mechanisms used by AR domain-containing proteins to recognize and bind substrates in the cell, as well as potential features of this binding pocket amongst other AR-containing proteins in similar protein families. This also opens the possibility of engineering new AR domain proteins with varying function based upon the novel discoveries of protein recognition and interactions that AR domain proteins partake in that could prove useful in drug development.

### 1.2. Ubiquitylation Signaling Pathway and Ubiquitin Chain Formation

The ubiquitylation signaling pathway (USP) involves a highly regulated enzyme cascade that results in the covalent attachment of ubiquitin on to a substrate protein [16]. First, the ubiquitin activating enzyme (E1) activates ubiquitin in the presence of ATP, allowing for the subsequent transfer of ubiquitin to the ubiquitin conjugating (E2) enzyme via a thioester bond. This in turn initiates the formation of an E2-E3 ubiquitin ligase complex that directs the transfer of ubiquitin onto a substrate protein to form a stable isopeptide bond between the *C*-terminus of ubiquitin and the ε-amine of a lysine residue on the substrate [16]. This post-translational modification is critical for regulating a wide array of biological processes including intracellular protein localization, the DNA damage response, protein activation or inactivation, innate immune response and 26S proteasomal degradation [17]. 

The human genome encodes for two E1 enzymes, 37 E2 enzymes, and hundreds E3 ligases allowing for exquisite specificity in substrate recognition for ubiquitin attachment [18]. There are three subfamilies of E3 ubiquitin ligases that are classified based on their structural and functional similarities—the really interesting new gene (RING), homologous to E6AP *C*-terminus (HECT), and RING-between-RING (RBR) E3 ubiquitin ligases [17]. The RING E3 ligases are the largest most well-studied subfamily with over 600 E3 ligases currently annotated in the human genome. The RING E3 ubiquitin ligases use their Zn^2+^-binding RING domain as a scaffold that engages and properly orients the E2~ubiquitin complex for efficient transfer of ubiquitin onto a substrate protein [19]. The HECT E3 ubiquitin ligases family is comprised of 28 members that all have a highly conserved bi-lobal HECT domain at their *C*-termini that contains an absolutely required cysteine residue for accepting ubiquitin from an E2 and the subsequent transfer on to its substrates [17]. HECT E3 ubiquitin ligases use their N-terminal protein–protein interaction domains for to carefully recognize and recruit proteins for ubiquitylation [17]. There are 14 identified members of the RBR E3 ubiquitin ligase subfamily that each contain a RING1-IBR-RING2 domain that employ a hybrid RING/HECT mechanism to attach ubiquitin on to its substrates [20]. Similar to the RING E3 ligases, the RBRs use their RING1 domain to recruit the E2~ubiquitin complex, which in turn is able to transfer ubiquitin onto its conserved catalytic cystine residue within the RING2 domain (aka Required for catalysis; Rcat) prior to covalently tagging its substrates with ubiquitin [21,22]. 

The specific attachment site(s) of ubiquitin on a substrate protein is decided by the E2-E3 pair [23], and depending on the ubiquitin chain type, the fate of the ubiquitin tagged protein is decided. For example, the attachment of a single ubiquitin (i.e., monoubiquitylation) can signal for various biological processes such as endocytosis, while ubiquitin chains (i.e., polyubiquitylation) connected by identical (homotypic) or varying (heterotypic/branched) linkers influence a myriad of signaling pathways yet can remain highly specific with each additional attachment. K48-linked polyubiquitin chain formation is the most well studied and understood modification that targets the substrate protein for degradation by the 26S proteasome [24,25]. Alternative polyubiquitin chains with different linkages have also been shown to regulate cellular processes. These include K6, K27, K29 and K63 polyubiquitylation to initiate the DNA damage response [26]; M1 and K11 polyubiquitin to activate the NF-κβ signaling pathway [26]; and various branched polyubiquitylation chains (i.e., K11/K48, K29/K48, K48/K63) have been observed to target proteins for proteolysis [27]. The complexity and permutations of ubiquitin chain types can include the modification of ubiquitin residues such as lysine acetylation that can repress K6 and K48 polyubiquitin chain formation [25], and serine/threonine phosphorylation that can act as an activator of the RBR E3 ubiquitin ligase parkin [28]. 

## 2. Ankyrin Repeats and the USP—Working Together to Regulate Intracellular Processes

**Figure 2:**
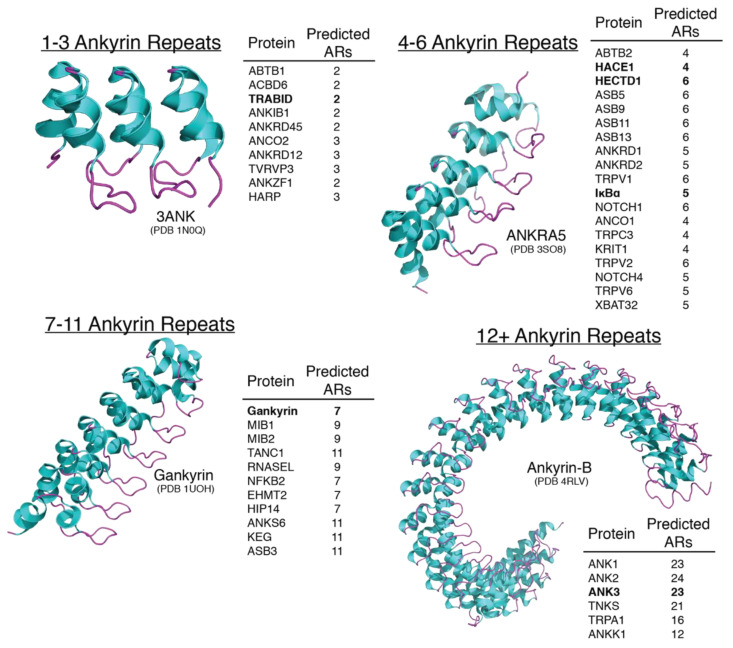
Examples of AR domain-containing proteins based on AR repeat number. AR domains can contain a wide variety of repeats, but predominately contain four to six. With each repeat, the 90° and L-shape formation becomes more prevalent resulting in a 3D revolving twist. In general, with each additional AR repeat, there is a correlation between complexity of the AR-containing protein’s biological role in cellular processes that it regulates. This may be due to the increased number in substrate binding pockets available that could be recognized by the substrate. The AR domain-containing proteins discussed in this review are highlighted in bold.

With the prevalence of AR domains in eukaryotic proteins (Figure 2) coupled with all of the reported intracellular processes regulated by ubiquitin, it not surprising that there are some important proteins and enzymes that take part at the poorly understood intersection of AR domains and ubiquitylation. This review aims to highlight the functional roles of some prominent AR domain containing proteins and what is currently known and unknown on their ubiquitin biology.

### 2.1. HACE1 and HECTD1: Ankyrin Repeat Containing HECT E3 Ubiquitin Ligases

**Figure 3:**
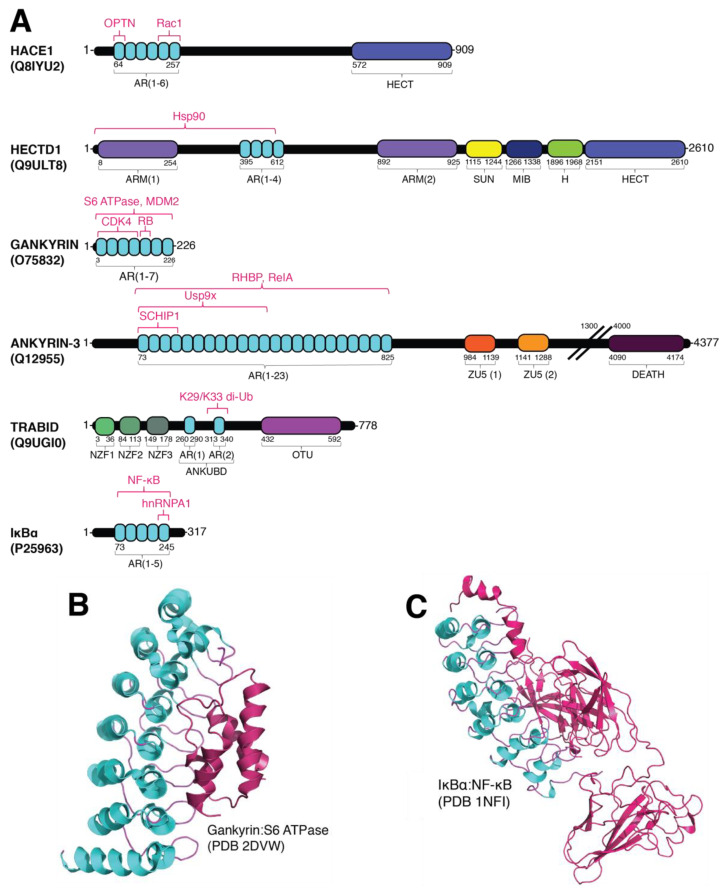
Domain architecture of AR domain-containing proteins and AR domain-complex formation. (**A**) Putative domain architecture of AR-containing proteins discussed in this review. While each protein has a variety of domains that facilitate protein–protein interactions, the AR domains are highlighted for their role in mediating the ubiquitylation signaling pathway. Domain abbreviations used include: AR, ankyrin repeat; HECT, homologous to E6AP *C*-terminus; ARM, armadillo repeat-containing domain; SUN, SAD1/UNC domain; MIB, mind-bomb domain; H, helical bundle; ZU5, ZU5 domain; DEATH, death domain; NZF, Npl4 zinc finger domain; OUT, ovarian tumor domain. Identified substrates and sites of interaction are highlighted in magenta. Substrate abbreviations used included: OPTN, optineurin [30]; Rac1, Ras-related C3 botulinum toxin substrate 1 [31]; Hsp90, heat shock protein 90 [32]; MDM2, mouse double minute 2 homolog [33]; CDK4, cyclin-dependent kinase 4 [34]; RB, retinoblastoma protein [35]; RHBP, rhodnius heme-binding protein [36]; RelA, transcription factor p65 [37]; Usp9x, ubiquitin specific peptidase 9 X-Linked [38]; proteosomal subunit S6 ATPase [39]; SCHIP1, schwannomin interacting protein 1 [40]; di-Ub, di-ubiquitin [41]; hnRNPA1, heterogeneous nuclear ribonucleoprotein A1 [42]; NF-κβ [43]. (**B**) Structure of AR-containing protein gankyrin in complex with S6 ATPase (PDB 2DVW; [39]). Gankyrin utilizes the internal b-hairpin loops to bind onto the *C*-terminal a-helix of S6 ATPase. (**C**) Structure of AR-containing protein IκBα in complex with NF-κB (PDB 1NFI; [43]).

Many HECT E3 ubiquitin ligases have been identified to have dual functions through their specific interactions with cellular proteins that is completely independent of their ubiquitylating activities. A prime example is HECT domain and ankyrin repeat containing E3 ubiquitin protein ligase 1 (HACE1), which was first identified in 2004 as a tumor suppressor caused by chromosomal translocation that leads to sporadic Wilms tumor with decreased HACE1 expression [29]. Apart from containing a highly conserved *C*-terminal HECT domain required for ubiquitylation, HACE1 was also the first member of the HECT E3 ubiquitin ligases to contain six predicted AR domains at its *N*-terminus (Figure 3A).

Besides being genetically linked to Wilms tumor formation, HACE1 has also been reported to play a role in the onset of liver, lymphoma, osteosarcoma, breast and colorectal cancers [44,45,46,47]. Studies have also revealed that HACE1 mediates Golgi membrane formation during cell division through the ubiquitylation of Rab proteins for proteolysis [48].

Various *N*-terminal putative protein interaction domains have been annotated for each HECT E3 ubiquitin ligase family member, which gave rise to the sub-classification of this family. This variety is predicted to be involved in substrate recognition and recruitment [17]. For instance, HACE1 has been reported to interact with a recently identified substrate optineurin (OPTN) through its AR domains (Figure 3A) [30]. The HACE1-dependent ubiquitylation of OPTN resulted in K27 and K48-specific polyubiquitin chains were observed to be covalently attached onto OPTN at K193 that can then promote the formation of an autophagy receptor complex with p62/SQSTM1. While K48 polyubiquitin chains are ideally utilized to signal for proteasomal degradation, K27 polyubiquitin chains can also be recognized by the proteasome but are rarely recognized and cleaved by deubiquitylases [49]. It is suggested that HACE1-dependent ubiquitylation of OTPN is required to encourage this complex formation, which in turn accelerates the elimination process of p62 leading to suppression of lung carcinoma cell growth [30]. 

HACE1 also acts as an adaptor protein that is critical in cardiac protection during hemodynamic stress. For example, HACE1 facilitates the transfer of ubiquitylated proteins p62 and microtubule-associated proteins 1A/1B light chain 3B (LC3) for autophagic degradation. This is accomplished through recognizing and binding to these modified proteins via its AR domain, independent of its typical E3 ligase activity [50]. HACE1 also possesses antioxidative stress response capabilities; it was previously reported that HACE1 promotes the activation of nuclear factor erythroid 2-related factor 2 (NRF2), a key regulator of the antioxidation stress response to mediate redox homeostasis through activation of antioxidative genes [51]. Decreased HACE1 expression levels were found to severely alter the expression levels and stability of NRF2, inhibiting antioxidative stress response precursors which eliminate oxidative triggers such as mutant Huntington protein [51,52]. Patients with Huntington’s disease (HD) have also been observed to have decreased HACE1 levels in the striatum, the region of the brain where HD initially manifests [51,52]. These cumulative results suggest that HACE1 can serve as an early stage neurodegenerative disease target. Improving our understanding on the mechanisms used by HACE1 to bind to its substrates through its AR domains is paramount as it remains unclear how exactly HACE1 utilizes its AR domain to modify these essential proteins. Further evaluation into this prominent domain within HACE1 can also potentially identify abnormalities in substrate recognition and binding the AR domain typically takes on, which can lead towards the development of neurodegenerative disease treatment.

Another intriguing yet poorly understood member of the HECT E3 ubiquitin ligase family is HECTD1. HECTD1 was first classified as a HECT E3 ubiquitin ligase in 2007 upon the discovery of its role in head mesenchyme development and neural tube closure [53]. Similar to HACE1, HECTD1 also contains AR domains in its *N*-terminus (Figure 3A). HECTD1 is also predicted through sequence similarity to have two ARMs that flank four AR domains, a SADL/UNC1 (SUN) domain, a mind-bomb (MIB) domain and a helical bundle that are located *N*-terminal to its HECT domain [54,55,56]. 

HECTD1 has been reported to play an important role in embryonic development by regulating cranial mesenchyme cellular behavior [53]. Researchers have shown that the *N*-terminal region of HECTD1 (ARM1 and the *N*-terminal AR domains; a.a. 1-551) was shown to interact with heat shock protein 90 (Hsp90) to influence cranial mesenchymal cell behavior (Figure 3A). Hsp90 is secreted from the cell to increase cellular motility, however, when HECTD1 targets Hsp90 via K63 polyubiquitin chain, this changes Hsp90’s intracellular localization which in turn inhibits its ability to be expressed extracellularly [32]. Interestingly, the boundaries for the predicted AR domains in HECTD1 should end at residue 612, where the third repeat ends at residue 491, whereas the fourth repeat starts at residue 579. This larger gap between AR domains 3 and 4 of HECTD1 is atypical, thus it can be speculated that either another folded domain or an unrecognized AR sequence may exist within this gap that might be required for Hsp90 recognition and binding. With HECTD1 also containing its first ARM repeat between residues 8-254, it is possible that Hsp90 could be recognized through a different domain. Further studies are needed to clarify the mechanism for HECTD1-dependent recruitment of Hsp90. 

HECTD1 plays an intricate role in regulating epithelial-mesenchymal transition (EMT) through ubiquitylation. EMT is an integral process that allows for plasticity in a phenotype switch in epithelial cells to mesenchymal cells [57]. The activation of EMT is essential during development, but it can also be triggered in various cancers through influencing cell-cell adhesion and metastasis [58]. HECTD1 contains eight nuclear localization sequences (NLS) and four nuclear export signals (NES), giving rise to its presence within the nucleus despite typical cytoplasmic localization; this localization is specifically mediated through exportin 1 (XPO1) [59]. With this ability to translocate from the cytosol to the nucleus, HECTD1 is able to regulate zinc finger protein SNAIL1 (SNAIL) expression levels. Since SNAIL increases mesenchymal characteristics in epithelial cells, HECTD1 controls SNAIL through ubiquitylation to targeting it SNAIL for cytoplasmic translocation and its subsequent proteasomal degradation. This translocation causes the repression of the tumor suppressor protein E-cadherin and helps to maintain proper EMT during development [60]. E-cadherin colocalizes to the cell membrane and encourages cell-cell adhesion which is believed to initiate tumor metastasis in its absence [61]. SNAIL regulates E-cadherin levels through binding at specific E-box sites on its promotor to inhibit expression [60,62]. Another EMT regulator protein, actin cross-linking factor 7 (ACF7), has been shown to interact with Rac1 to stabilize membrane protrusions while simultaneously increasing motility [63]. HECTD1 was also identified to regulate EMT through targeting ACF7 for proteasomal degradation. A negative correlation was observed between the expression levels of both HECTD1 and ACF7 in breast cancer cells that further encouraged metastasis and cytotoxin drug resistance due to the inability to readily target ACF7 for degradation [63]. Since histone methyltransferase interacts with SNAIL through its AR domain to regulate EMT [64], as well as ACF7 having multiple protein–protein interaction domains [65], it can be speculated that HECTD1 regulates EMT by its AR domains to recognize and bind to both SNAIL and ACF7 to target these proteins for degradation. 

Beyond EMT, HECTD1 has also been shown to play an essential in regulating Iκβα, a key regulator of the NF-kB transcription factor. Specifically, HECTD1 mediates Iκβα K48 polyubiquitylation through its direct interaction with the ribosomal protein subunit 3 (Rsp3). HECTD1 forms a ternary complex with latexin (LAX), Iκβα and ribosomal protein subunit 3 (Rps3); this triggers Iκβα ubiquitylation and upregulates the NF-κβ stimulated inflammatory stress response [66]. Interestingly, only Rsp3 detachment from the complex attenuates LEX/HECTD1 interaction [66]. Overall, the formation of the Rsp3/HECTD1 complex encourages ubiquitylation of Iκβα and LAX can competitively bind to the Rsp3 binding site to inhibit ubiquitylation. Further biochemical and biophysical analysis are needed to further clarify how HECTD1 uses its AR domain for substrate recognition and subsequent protein degradation by the proteasome. 

### 2.2. Usp9x and TRABID: Deubiquitylases Facilitating Ub Cleavage through Internal and External AR Domains

Deubiquitylation involves the enzymatic removal of ubiquitin or ubiquitin chains from a substrate protein, and arguably plays the most critical role in regulating ubiquitylated substrate fate [67]. While the human genome encodes for ~90 deubiquitylases (DUBs) [67,68], their biological relevance beyond deubiquitylation is still unclear. Recent studies indicate that DUBs may be an ideal drug target [69] through enhancement or inhibition of substrate binding [70]. 

Ubiquitin specific peptidase 9 X-linked (Usp9x) is a prominently known for acting as the DUB to protect SMURF1 and RNF115 from proteolysis [71,72], and there is emerging evidence that Usp9x can also interact with other AR-containing proteins to regulate intracellular events. For instance, Usp9x has been observed to interact with the cytoskeletal protein ankyrin-3 (ANK3) to promote spine morphogenesis [38,73]. Studies have shown that the disruption of Usp9x and ANK3 complex formation results in deficient synaptic structural maintenance that can contribute to neurodevelopmental disorders [38]. Usp9x/ANK3 complex formation is stimulated by TFG-β signaling leading to the phosphorylation of Usp9x, which in turn increases Usp9x binding affinity to the AR repeats of ANK3 [73] (Figure 3A). Interestingly, abnormal expression of Usp9x and ANK3 have also be correlated with neurodevelopmental and neurodegenerative and psychiatric disorders such as autism-spectrum disorder, Parkinson’s disease, Alzheimer’s disease, epilepsy, and bipolar disorder [73,74,75,76]. These cumulative reports suggest that AR domain-containing proteins are clinically relevant in various diseases and the USP through E3 ubiquitin ligase protection. Further investigation should be directed towards how other DUBs utilize their own or external AR domains to facilitate USP. 

Intriguingly, TRAF-binding domain (TRABID) was found to contain two AR domains between a ubiquitin binding domain, named AnkUBD [41], which remained hidden due to the elusive characteristics of its AR domain (Figure 3A). TRABID has been suggested to play a role in the Wnt signaling pathway by using its AnkUBD domain to properly orient heterotypic polyubiquitin chains to specifically cleave K29 and K33 polyubiquitin linkages (Figure 3A) [15]. TRABID has also been shown to regulate the expression levels of the transcriptional regulator Twist1 in hepatocellular carcinoma (HCC). TRABID is responsible for the specific cleavage of K63-linked polyubiquitin chains from Twist1 resulting in Twist1 being able to form a complex with beta-transducin repeats-containing protein (β-TrCP) and the subsequent K48-specific polyubiquitylation of Twist1 for proteasomal degradation [77]. With the emerging evidence that DUBs and AR-containing proteins could serve as novel drug targets, it is imperative that increased attention be directed towards improving our knowledge on how AR proteins are involved in protein–protein interactions and intracellular targeting.

### 2.3. Gankyrin: An Oncogenic AR Domain-Containing Protein

Gankyrin is a seven AR domain-containing liver oncoprotein that is involved in a myriad of cellular processes including cell cycle progression, liver regeneration, protein translocation and enzymatic regulation (Figure 3A) [78,79,80]. When comparing the primary sequences of gankyrin’s seven AR repeats there is significant conservation within the AR repeats especially between the first six AR repeats (Figure 3A,B). Previous studies have shown that the overexpression of gankyrin is linked to the onset of various malignancies making it a potential oncogenic biomarker [78,81]. It has also been suggested that gankyrin competes for cyclin-dependent kinase 4 (CDK4) binding with INK4a in order to regulate transcription factor e2f expression (Figure 3A) [80,82]. 

Importantly, gankyrin plays a critical role in the encouragement of mouse double minute 2 (MDM2), a RING E3 ubiquitin ligase, to ubiquitylate p53 for cytoplasmic localization and degradation [83]. Specifically, gankyrin utilizes its AR domain to bind to MDM2 to facilitate the MDM2-dependent ubiquitylation of p53 (Figure 3A) [83]. Residue deletions within the gankyrin AR domain abolished gankyrin/MDM2 interactions but did not affect MDM2/p53 interactions, suggesting that gankyrin associates with MDM2 away from the p53-MDM2 binding site. Evaluating AR-containing proteins in comparison to gankyrin can reveal potential ubiquitylating mediating functions. 

Gankyrin also has been demonstrated to be involved in EMT through its regulation of downstream cytokines interleukin 6 (IL-6) and transforming growth factor beta (TGF-β) that induce the EMT phenotype [84]. In contrast to HECTD1, increased gankyrin expression levels was observed to cause decreased E-cadherin expression in non-small cell lung cancer (NSCLC) cells that overexpress gankyrin [84]. 

The mechanisms used by gankyrin to regulate transcription factor levels is currently unclear and need further examination. Likewise, a detailed comparison of gankyrin to a similar AR domain-containing protein that regulates nuclear factor-kappa-β (NF-κβ) expression levels is warranted. For instance, the Iκβα is a five AR-containing regulatory protein with capabilities of regulating NF-κβ expression levels (Figure 3A,C). Once Iκβα is modified via ubiquitylation and phosphorylation, it is released from the p50/RelA (p65) complex to allow for nuclear localization and NF-κβ activation, followed by ubiquitin-mediated degradation [78,85]. Similarly, gankyrin was discovered to regulate NF-κβ through its AR domain (Figure 3A) [42]. Gankyrin was observed to bind to and inhibit the NF-ĸβ/RelA complex activity by preventing NF-ĸβ nuclear localization [78]. Similar methods should be evaluated when identifying other AR domain-containing proteins that play similar roles in various signaling pathways to further characterize and better understand the significance of the unique AR domain. 

## 3. AR Domain-Containing Proteins with Unknown Ubiquitylation Mechanisms—What Is Next?

AR domain-containing proteins are ubiquitous and play essential roles in numerous biological processes that can also influence the onset of various diseases and disorders. Many AR domain-containing proteins have been identified in numerous occasions to support the USP and reverse the process of deubiquitylation through sequence alignment. Being essential scaffolds to mediate protein–protein interactions, it is intriguing that this domain is not functionally driven but rather dependent on the structural characteristics of the AR domain. Understanding how these AR-domain containing proteins and their role in ubiquitin signaling on the molecular level is a major challenge in our present understanding of their function(s) and activity. Expanded biochemical and biophysical examinations on the novel mechanisms used for AR-domain protein recruitment are needed. By experimentally identifying and validating potential substrates and interactors for these AR-domain proteins, we will be able to improve our knowledge of how these proteins work and will be able to better assess if these proteins could serve as novel drug targets or as biomarkers for disease.

## Data Availability

All figures and discussed literature are found in the main text of this review.
